# Individual and environmental factors influencing the dietary behaviour of healthcare workers during night shifts in the Netherlands: a qualitative study

**DOI:** 10.1017/jns.2025.10041

**Published:** 2025-10-10

**Authors:** Fleur van Elk, Karen M. Oude Hengel, Coen Dros, Alex Burdorf, Heidi M. Lammers-van der Holst

**Affiliations:** 1 Department of Public Health, Erasmus University Medical Center, Rotterdam, The Netherlands; 2 Department of Work Health Technology, Netherlands Organisation for Applied Scientific Research TNO, Leiden, The Netherlands

**Keywords:** Behaviour change model, Dietary behaviour, Environmental level, Individual level, Night workers, EnRG, Environmental Research framework for weight Gain prevention at environmental level, B, Barrier, F, Facilitator

## Abstract

This qualitative descriptive study aimed to explore dietary habits among healthcare workers during night shifts and to identify individual and environmental factors that influence their dietary behaviour during night shifts. Individual semi-structured interviews were conducted with twenty-five healthcare night female workers, which were recruited via email invitations from managers and posters placed in central workplaces at a university medical centre in the Netherlands. The interview protocol was developed following an integrated behaviour change model focusing on individual (I-Change model, i.e., awareness, motivation, intention, and ability) and environmental (Environmental Research framework for weight Gain prevention at environmental level (EnRG), i.e., physical, policy-related, economic, and sociocultural) factors. Inductive analyses were conducted to explore dietary habits, while deductive thematic analysis was applied to identify potential factors influencing dietary behaviour. Female healthcare workers in night shifts generally made poorer dietary choices during night shifts than during other shifts. Seven key themes were coded for dietary behaviour. Based on the domains of the integrated behaviour change model, four individual and five environmental key themes were established, within which 41 sub-themes were coded. Key individual factors included awareness (i.e., lack of knowledge about timing and type of consumption) and motivation (i.e., attitude and efficacy to eat healthy). Critical environmental factors included physical and sociocultural work environment, organisation of work, and lack of organisational policies. To conclude, future dietary interventions for healthcare night workers should target both individual behaviours and the workplace environment, with an emphasis on raising awareness and enhancing organisational policies to promote healthy dietary habits.

## Introduction

Working during the night regularly results in a sleeping pattern that is desynchronised with the natural rhythms of our biological clock,^(1)^ and may lead to irregular feed-fast cycles.^(2)^ The circadian system generates approximately 24-hour rhythmicity regulating multiple bodily functions, such as hormone secretion, appetite, and temperature. The system is organised hierarchically with a central clock in the suprachiasmatic nucleus of the brain and a number of peripheral clocks in most organs of the body.^(3)^ Natural light exposure is the most important timing cue (zeitgeber) for the central clock, and feed-fast cycles are important timing cues for the synchronisation of the peripheral clocks. Therefore, alterations to the timing, amount, and composition of food intake can affect the synchronisation of the circadian system.^(4)^

Circadian desynchronisation has been associated with adverse metabolic consequences.^(5)^ This is shown in the association of night shift work with increased risks of overweight and obesity,^(6)^ metabolic syndrome,^(7)^ and type 2 diabetes mellitus.^(8)^ Night shift work is also associated with changes in the timing, quantity, and quality of food intake.^(9)^ A recent systematic review showed that night workers consume more soft drinks and products containing saturated fat, and skip more meals than day-time workers.^(9)^ Another systematic review showed that shift workers have more irregular and frequent meals, increased snacking/eating during the night, and they consume fewer core foods and more discretionary foods than day workers.^(10)^ Changes in dietary behaviours (e.g., timing and choice of food intake) and the resulting circadian desynchronisation may therefore be a major contributor to the increased risk of metabolic diseases among night workers.

The increased risks of unhealthy dietary behaviour and metabolic diseases due to working night shifts highlight the importance of developing effective interventions for night workers to improve their dietary behaviours at night. Whereas our body is not built to be awake and eat during the night, and it is recommended not to eat at night to prevent internal circadian misalignment,^(11)^ most night workers consume nutrients during the night to have enough energy to perform their work. Research on eating patterns among police officers showed that overall no differences in caloric intake between shift types were present, however when looking at the hours during the night (11:00 PM–6:00 AM), more calories were consumed on night-shift days than on other shifts or days off, and the intake occurred significantly later for days with night shifts compared with days off.^(12)^ Even though no consensus exists on the recommendations for what and when it is best to consume during the night, more general dietary guidelines for shift workers are in place.^(13,14)^ These include the advice that workers need to follow the normal day and night dietary pattern as much as possible, divide their 24 h food intake into three meals, and eat small portions when eating during the night shift. Furthermore, it is recommended to consume vegetables, wholegrain products, and relatively high-protein products, while avoiding convenience foods, high-carbohydrate foods, non-fibre carbohydrate foods, and sugar-rich foods and drinks.^(13,14)^

To successfully develop and implement effective nutritional interventions for night workers, more insight is needed on behavioural patterns of healthcare workers during nights. This is important as there is a lack of information on the extent of healthy or unhealthy dietary patterns in this group. Additionally, it is important to gain insight into the factors that hamper or facilitate workers towards healthy behavioural patterns during night shifts. A previous study showed that a complex set of barriers and facilitators influence the dietary behaviour of shift workers.^(15)^ Thereby, it is important to detect factors at both individual (e.g. preferences) and environmental level (e.g. opening hours canteen, availability of healthy food, working conditions), because both factors are important to achieve a change in behaviour.^(16)^ Until now, these influencing factors have mainly been understudied or underrepresented in most studies on chrononutrition among night workers. However, insight in the influencing factors at individual and environmental level are needed to better develop and implement intervention at all levels. Qualitative studies are an adequate way to gather nuanced and detailed information about the target group’s perspective. Therefore, the current study used a qualitative approach by integrating and using behaviour change models that focus on individual as well as environmental factors influencing dietary behaviours at night.

Against this background, the current study aimed to explore i) the dietary habits during night shifts among healthcare workers, and ii) which individual and environmental factors influence their dietary behaviour during night shifts by using a qualitative research design.

## Methods

### Study design, population and setting

A qualitative study consisting of theory-based semi-structured interviews was conducted among 25 healthcare workers (i.e., physicians, nurses or students) with night shifts in a university medical centre in the Netherlands. We adopted an interpretivist epistemological standpoint to explore participants’ experiences and perceptions in the context of working at night. By conducting interviews with pre-defined prompts (open-ended questions) and follow-up probes we gained a deeper understanding and participants could further nuance their responses. Participants were recruited purposively with a digital invitation via managers and a targeted display of posters at central workplaces in the hospital. After expressing their interest by sending an e-mail to the project group, one of the researchers planned an online or face-to-face interview with the participant, depending on his/her preference. Inclusion criteria were working at least one night shift in the three months before the interview and having a command of the Dutch language. Participants gave written informed consent to participate and to audio record the interview, and they were compensated for their time with a gift card. Interviews were conducted until no new information was gathered (i.e., data saturation).

The organisation in which the study took place was a university medical centre in The Netherlands, with over 16,000 employees. The organisation has two in-house restaurants with catering facilities and multiple cafés which are open until 7:00 PM at the latest. Several departments have a ‘patient kitchen’, where food for patients is stored and prepared. One supermarket is located in the hospital and is open from 7:00 AM until 8:00 PM on weekdays (9:30 AM until 6:00 PM on Saturdays; 10:00 AM until 5:00 PM on Sundays). Seven healthy fridges with vegan options are located around the hospital. All facilities are accessible for employees and patients. During the study period, vending machines with snacks and sodas were being phased out, but such machines were still present at specific locations. The Medical Ethical Committee of Erasmus MC declared that the Medical Research Involving Human Subjects Act does not apply to the study (MEC-2023-0156). The study was conducted according to the principles of the Declaration of Helsinki (version 64, October 2013), Code of Conduct for Health Research 2022.

### Materials

**Fig. 1.**
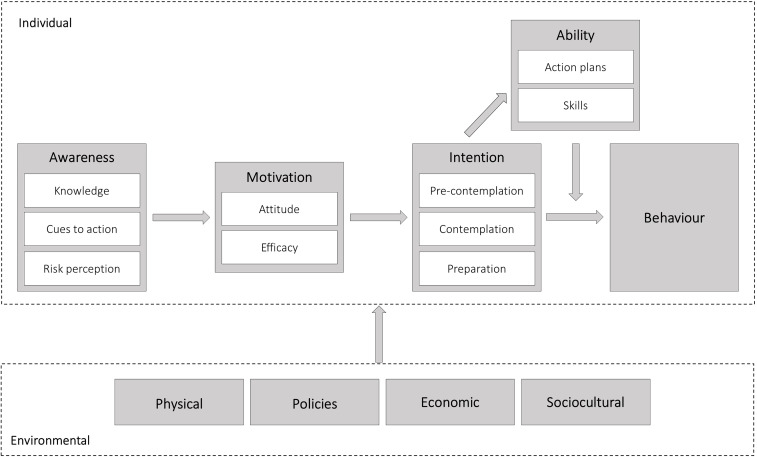
Integrated behaviour change model, based on the Integrated Model for Explaining Motivational and Behavioural Change (I-Change model, see upper part)^(17)^ and the Environmental Research framework for weight Gain prevention (EnRG, see lower part)^(18)^.

An interview protocol was developed, which consisted of one part with questions about the typical dietary pattern during nights shifts, and another part about individual and environmental factors influencing this pattern. These latter questions were based on the integrated behaviour change model that was created for the current study (Fig. 1). Whereas most existing behaviour change models focus solely or predominantly on either individual or environmental factors, our model integrates both levels as both are considered important for comprehensive interventions.^(16)^ We combined the Model for Explaining Motivational and Behavioural Change at individual level (I-Change model)^(17)^ and the Environmental Research framework for weight Gain prevention at environmental level (EnRG).^(18)^ The I-Change model focuses on individual factors, which are i) awareness (consisting of knowledge, cues to action, and risk perception), for instance knowing the health risks of unhealthy eating during night shifts, ii) motivation (consisting of attitude and efficacy), such as the perception of the capability to change dietary behaviour during night shifts, iii) intention to change dietary behaviour (consisting of the pre-contemplation, contemplation, and preparation phases), and iv) ability (consisting of action plans and skills), for instance, making concrete plans to actually change dietary behaviour. The environmental part of the model in the current study includes i) physical (e.g., food available at night), ii) policies (in the organisation concerning food), iii) economic (e.g., the price of healthy food), and iv) sociocultural (e.g., the influence of dietary behaviours of colleagues). When integrating the existing models into our final model, we omitted the I-Change model factor ‘social influence’, as this overlaps with the ‘sociocultural’ environmental factor of the EnRG model, which encompasses a broader sense of sociocultural influence.

The interview protocol was pilot tested with one physician and one nurse to ensure the questions were clear, and the protocol was optimised by reformulating and adjusting some questions.

### Data collection

All interviews were conducted face-to-face in a private setting at the hospital and it took on average 30–40 minutes per interview. Fifteen interviews were conducted by a female PhD student (FvE, MSc) and 10 interviews by a male final-year medical student (AR, BSc). No relationship existed between the interviewer and the interviewee prior to the study. Participants were aware of the general goal of the research, namely to gain insight into factors influencing their dietary behaviour. The interviews were audio recorded, manually transcribed non-verbatim by the researcher who conducted the interview, and pseudo-anonymised. No field notes were made during or after the interviews.

### Analysis

Two researchers (FvE and CD) read all interview transcripts and generated a codebook for the second research question based on the factors at individual and environmental levels from the model (Fig. 1). The codebook consisted of the definition of the codes and sample quotes from the interviews. FvE and CD both coded the first four interviews in NVivo (QSR International Pty Ltd) to reach a consensus on definitions, and thereafter the remaining interviews were divided between the two researchers for coding. The coding process was regularly discussed, and difficulties and discrepancies were discussed until a consensus was reached. If no consensus was reached, two other researchers were consulted (KOH and HLH). For the transcript data about the dietary habits during night shifts, inductive thematic analysis was performed, meaning that codes were generated based on the available data. For the transcript data about individual and environmental factors, deductive analysis was conducted, meaning that the data was coded according to the pre-defined codebook based on our model. In the latter case, additional inductive thematic analysis was performed in which similar responses within each factor of the model were grouped to find the themes that summarised the role of that factor in influencing dietary behaviour. When no new themes appeared, authors FvE and CD agreed that data saturation had been reached.

## Results

### Characteristics of participants

A total of 25 healthcare workers participated. All participants were female, with a mean age of 30.44 (SD: 8.12). The majority was nurse (76%), and participants had worked on average 8.56 (SD: 8.75) years on night shifts and 5.28 (SD: 2.94) night shifts per month. Most participants worked nine-hour night shifts, which started between 10:30 and 11:00 PM and ended between 7:30 and 8:00 AM, and a few worked nine-hour shifts as well as twelve-hour shifts, which started between 7:30 and 8:00 PM and ended between 7:45 and 8:00 AM. Participant characteristics are summarised in Table 1.

**Table 1.** Demographic and work characteristics of hospital workers with night shifts (n = 25)

	n (%)
Age (in years)	
20 < 30	14 (56%)
30 < 40	8 (32%)
40 < 50	–
50 < 60	2 (8%)
Gender	
Female	25 (100%)
Male	0 (0%)
Occupation	
Nurse	19 (76%)
Physician	1 (4%)
Dual job (nurse and researcher/trainer)	3 (12%)
Medical student^a^	2 (8%)
Years with night shifts	
0 < 5	9 (36%)
5 < 10	9 (36%)
10 < 15	3 (12%)
15 < 20	1 (4%)
≥ 20	2 (8%)
Night shifts per month	
1 < 5	14 (56%)
5 < 10	8 (32%)
10 < 15	3 (12%)

a Medical students worked under nurse or physician supervision during irregular hours, either as medical interns or in side jobs.

### Dietary behaviour

We clustered the responses from healthcare workers into 7 key themes, mainly concerning participants’ timing and type of food and drinks during night shifts versus other shifts. Most participants primarily had a similar dietary pattern or routine during each night shift. Compared with day and evening shifts, the majority reported eating more unhealthily (e.g., snacking more, drinking less, bigger portions), while some mentioned eating more healthily during night shifts. Although the specific times workers consumed which products varied from one night worker to another, the eating periods were generally i) at dinner time before the night shift, ii) at the beginning of the shift, iii) in the middle of the shift between 2:00 AM and 5:00 AM, or iv) at the end of the shift. Some participants mentioned eating ‘when they want to’ without having a regular schedule. The majority of the participants brought food from home and some participants also got food from the patient kitchen.

The majority ate a regular warm meal around dinner time before their night shift, whereas a few participants ate a warm meal during their night shift. Other products consumed by the majority were fruit and yoghurt with toppings (e.g., fruit, muesli/granola, nuts) at the end or after the shift. Crackers and bread were also mentioned regularly, with variations in toppings or spreads and timing of consumption. Additionally, salads, vegetables, and nuts were frequent choices, mainly consumed in the middle of the shift. Generally, participants ate protein-rich products, although many also indicated to consume fatty or sugar-/carbohydrate-rich snacks (e.g., biscuits, chocolate, grilled sandwich, salty snacks) sometimes or regularly, mainly at the start of the shift or around 4:00 AM.

Concerning drinking at night, some night workers consumed only water and tea, whereas most drank coffee as well. Of those drinking coffee, most participants drank 1 or 2 coffees, while a few drank 3 or more. Coffee was mostly consumed at the beginning of the shift, while others consumed coffee until 3:00–4:00 AM. Some night workers consumed other drinks such as soft drinks, energy drinks, and smoothies.

### Individual factors influencing dietary behaviour

Several individual factors were identified across the model that influenced dietary behaviour, with the exception of ‘skills’ within the ‘ability’ domain. The domains from the integrated behaviour change model were applied as 4 key themes. Table 2 summarises 22 sub-themes of barriers and facilitators in relation to healthy dietary behaviour during night shifts in each individual domain and sample quotes from the interviews are provided.

**Table 2.** Barriers and facilitators at individual level for healthy dietary behaviour during night shifts among hospital workers (n = 25), for each individual domain in the integrated behaviour change model

Domain	Factor	Number of participants reported	Theme	Barrier (B)/facilitator (F)	Example quotes
Awareness	Knowledge	24	1. Type of food	B and F	*‘At some point I have read that you should eat protein-rich products and not too many fast sugars in order to not have spikes and dips in blood sugar levels.’ –* participant F
			2. Timing of food consumption	B and F	*‘In principle, I know that when you go in [the night shifts], you should go with [the pattern of] your family, and I try that. You should not eat too heavily at night. I just don’t know well what the best timing is to actually eat.’ –* participant Y
			3. Type of drinks	B and F	*‘[You should drink] at least not too many refined sugars, not too much, no soft drinks, also no coffee.’ –* participant W
			4. Timing of drinking	B and F	*‘I think you have to, I don’t know what time, but don’t drink too much coffee. Especially not before you go to sleep during the day.’*- participant X *‘I do not have much knowledge [about timing of drinking] to be honest.’ –* participant P
			5. General knowledge on dietary behaviour during night shifts	B and F	*‘Well, especially [knowledge about] keeping yourself fit and sharp. And because your body is already disturbed by the change in day-night rhythm, you should consume the right nutrients to stay fit, also for the job.’ –* participant I *‘I know a fair amount about healthy eating in general, but when it comes to eating during the night, I actually know very little. So, I go a bit by my intuition. In terms of knowledge, I really don’t know much about it.’ –* participant B
	Cues to action	2	1. Being exposed to unhealthy others	F	*‘I think it’s more unconscious. Because I have many patients with vascular diseases and kidney conditions, I think, “I don’t want to end up like that.” This leads me to make better choices. It’s more in my unconscious mind rather than something I consciously think about.’ –* participant J
	Risk perception	9	1. Risks to health	F	*‘I think that in the long term, it [healthy eating at night] is simply better. I don’t think night shifts are really good for your health, and I believe that if you eat very poorly during the night, it makes things even worse.’ –* participant N
			2. Preventing risks to health	F	*‘So I think you can’t prevent everything, but I believe you can prevent quite a lot if you don’t also have an extra stress factor, and a healthy diet also has an impact on it.’ –* participant M
Motivation	Attitude	25	1. Influence on mental and physical health	B and F	*‘[When I eat healthily at night] I just feel fit, I can get through the night shifts well, I sleep well, and even after my night shift, when I wake up and eat at 6:00 PM, I still have the energy to exercise before starting my next night shift. I think those are definite advantages.’ –* participant H
			2. Influence on sleep	B and F	*‘I notice that before, I used to lie awake because of stomach issues, and now [that I eat more healthily at night] I just fall asleep and wake up 8 hours later. That’s quite a significant improvement.’ –* participant F
			3. Rewards and preferences	B	*‘I think you always intend to eat healthily. But then you sit at work at 4 AM, and you already feel sorry for yourself for having to be there, so you don’t have much trouble saying, ‘Okay, I’ll eat that pizza anyway’.’ –* participant D *‘Well, it [healthy food] is not always your first choice, so to speak. It is your first choice in your head, because you know it’s better.’ –* participant T
			4. Investment of time and effort	B	*‘Sometimes it [healthy food] takes more time to prepare. Eating a muesli bar or a sandwich is quicker than peeling an apple, peeling a pear, cutting a cucumber into pieces, which also dries out very quickly. So I think it’s less convenient. It takes a bit more effort.’ –* participant A
			5. Influence on functioning at work	F	*‘I notice that healthier eating helps me to concentrate better at work than when I just snack and get those sugar spikes, and then crash again.’ –* participant T
	Efficacy	25	1. Needs and appetite	B and F	*‘Well, you just get hungry more often [at night]. And then unfortunately, you don’t crave very healthy things. We were talking about that today, too. If only we craved broccoli or something healthy. But no, it’s more like a nice piece of chocolate or a bit of cheese here and there. So yeah, I find that quite difficult.’ –* participant P
			2. Mental and physical health status	B and F	*‘You know what it is, mentally you’re not strong at all [at night]. During the night shifts, I’m emotional, I’m tired, it’s very difficult for me to motivate myself to do things, my house is a mess, I don’t tidy up, I feel lethargic. I have very little energy.’ –* participant S
			3. Temptations and discipline	B and F	*‘Once I start eating [unhealthily], well then I kind of, I don’t know. I’m generally an easy eater, so if there’s something there or if I bring something, I won’t resist. I won’t start. I don’t feel the need. But if it’s there and I start, then it’s a different story.’ –* participant V *‘Due to the night shifts, you also get a kind of blow, and you don’t really have willpower, I notice that too, the willpower to, for example, turn something [food] down. It’s much easier to say ‘come on!’’ –* participants B
			4. Habits	B and F	*‘I think it’s also a habit, you know. I think, once you start taking something tasty to work, then you always think, ‘Oh, I’m on a night shift, so I need to grab something good.’ If you don’t do that, then it’s not in your system.’ –* participant V
			5. Responsibilities at home and living situation	B and F	*‘I have a family with children. Especially since having children, we’re conscious of healthy eating. So in that sense, it indirectly affects my diet during night shifts as well.’ –* participant X *‘I live alone, so I have to manage everything myself, which can be challenging at times. You often have to do everything at the same time. When you live with someone, they might have already prepared the meal, that makes a difference.’ –* participant K
			6. Preparation at home	B and F	*‘I always try to do my grocery shopping before my night shifts. But if I haven’t done it, or if my husband hasn’t done it, then I just take what I have at home, and that is not always very healthy. Sometimes I take less because I don’t have it at home and don’t have time to go to the supermarket. So yes, that also has an influence.’ –* participant P
			7. Reasons not to prepare at home	B and F	*‘Mainly, lack of time prevents me from bringing something from home or preparing something at home. When you’re sleeping and wake up, for example, at 6 PM, it’s often tight [in terms of time] to go to the store, cook, and take care of things at home.’ –* participant O
			8. The weather	B and F	*‘And especially in the winter I’ve experienced that. I’ve only been through one winter [since I’ve been working here], and you tend to crave something warm in the evenings. So you end up ordering something in or bringing something with you, often a ready-made meal.’ –* participant M
Intention	Intention	7	–	–	*‘Well, when you talk about it like that and think about it, it does make me realise it [eating healthily at night] is important. Then I think, ‘Oh, I should also be healthier.’ But knowing myself, I also know that next week, when I’m on night shifts again, it will probably be less likely [that I’ll eat healthily].’ –* participant O
Ability	Action plans	4	1. Making action plans	F	*‘I’m also working in a new department, so I want to try to get it right from the start and not let the patterns from the previous department creep back in.’ –* participant C
	Skills	0	–	–	–

#### Awareness

All but one participant (n = 24) reported on whether they had adequate knowledge regarding diet during night shifts. Most participants made statements about general knowledge concerning diet at night, whereas some participants stated that they lacked any knowledge. Evidence-based knowledge was mentioned by some, such as the importance of maintaining the biological rhythm concerning dietary intake, the difference in digestion and insulin levels at night, and the influence of diet at night on physical fitness, sleep, and performance. Concerning the composition of food at night, the majority of night workers felt they had some specific knowledge. In line with recommendations, the majority knew that high-protein food is preferred to high-carbohydrate and fatty food. It was also mentioned that fruit and nuts are healthy at night, although recommendations state that timing of consumption determines whether this is healthy. Most night workers had limited or no knowledge on the timing of food consumption at night, as only a few knew the recommendation to consume relatively more protein at the beginning and in the middle of the shift, more carbohydrates at the end of the shift, and to eat breakfast before day-time sleep. While a few participants thought it is best to eat only at the beginning or the end of the shift, some others assumed consumption of food should be spread out. Regarding drinks, night workers were generally aware that water and non-caffeinated tea were preferable to sugar-rich or carbonated soft drinks and caffeine. Most participants assumed caffeine should be avoided, whereas some others thought caffeine intake should be limited to the beginning of the shift.

Some participants (n = 9) expressed awareness of the risks of an unhealthy diet at night. They reported, for example, the risks of overweight and cancer, and the effects on driving home safely after night shifts.

Cues to action were rarely addressed (n = 2). A few participants mentioned being exposed to sick patients and unhealthy colleagues as cues to maintain a healthy diet at night.

#### Motivation

All participants (n = 25) reported on their attitude towards healthy dietary behaviours during night shifts in terms of experienced advantages and disadvantages of the behaviour. All participants mentioned the positive influence of healthy dietary behaviour during night shifts on their physical or mental health. Most workers experienced a better balance in energy during the shift when eating healthy. Moreover, the majority were motivated to eat healthily at night, because it made them feel fitter, and because they experienced negative effects of eating unhealthy food (e.g., high-fat foods), such as abdominal complaints (e.g., stomach pain, bloating, and heartburn). Maintaining the biological rhythm and a healthy weight were mentioned by a few participants as motivators for healthy eating at night. Half of the participants also experienced the advantage of sleeping better when they eat a healthy diet or have some food at the end of the night shift, while some mentioned the negative influence of coffee on their day-time sleep. The work itself was also influenced, as some night workers commented on the advantage of a healthy pattern as it increases concentration and helps them stay awake and get through the night shift. Some barriers to adopting a healthy diet at night were also mentioned. These included rewarding oneself during the night shift with snacks, the preferences for unhealthy products at night, and the time and effort needed to prepare healthy food beforehand.

All participants (n = 25) commented on their perceived capability to adhere to a healthy diet at night (i.e., efficacy). Needs and appetite at night were mentioned as barriers to healthy dietary choices by some participants, as they experienced more cravings for unhealthy snacks. Other barriers were the need for caffeine and sweets to stay awake, the effects of the mental and physical state on unhealthy choices at night (e.g., tiredness, hormonal influences, and negative state), and the inability to resist the temptations of unhealthy snacks at night. The home situation also influenced eating habits, as almost half of the participants indicated that they were prone to bringing unhealthy products to work when they had not prepared food at home. Barriers to preparing food were a shortage of time or energy for buying groceries or the preparation itself, mainly because of the day-time sleep between night shifts. However, some participants indicated that time management at home was not an issue in preparing food and therefore in healthy eating during night shifts. Furthermore, a few found it more difficult to adhere to a healthy dietary pattern at night during winter. Facilitators concerning efficacy were that some participants were able to resist temptations at night or experienced minimal effort in maintaining a healthy diet when a healthy night routine is established. Few night workers mentioned a low appetite at night, not liking snacks, or their dietary restrictions as facilitators. Moreover, some reported that their responsibilities at home and the living situation positively influenced their dietary behaviour. To illustrate, setting a good example for their children was a facilitator for healthy eating during night shifts.

#### Intention

Only some night workers (n = 7) reported specifically on the intention to change their behaviour to a healthier dietary pattern at night. However, the different phases of intention (i.e., pre-contemplation, contemplation, and preparation) were not asked about explicitly.

#### Ability

Specific action plans to change dietary behaviour during night shifts were rarely explicitly addressed (n = 4), except for a few workers trying to write down what and when to eat or changing the dietary pattern at the start of a new job in another department. Moreover, night workers did not comment on their skills regarding dietary behaviour during night shifts.

### Environmental factors influencing dietary behaviour

Table 3 shows that factors in all four environmental domains of the model (physical, policies, economic, and sociocultural environment) were reported to influence dietary behaviour. Table 3 summarises 19 sub-themes of barriers and facilitators in relation to healthy dietary behaviour during night shifts in each domain, and sample quotes from the interviews are provided. One additional environmental domain also emerged from the data, namely the organisation of work, as the model was not sufficient to integrate these factors into one of the existing environmental domains, resulting in 5 defined key themes. All participants (n = 25) reported on each environmental domain, except for the economic environment (n = 24).

**Table 3.** Barriers and facilitators at environmental level for healthy dietary behaviour during night shifts among hospital workers (n = 25), for each environmental domain in the integrated behaviour change model

Domain	Number of participants reported	Theme	Barrier (B)/facilitator (F)	Example quotes
Physical	25	1. Availability of food for employees provided by the organisation	B and F	*‘We do get fruit in the department, but it’s gone within a day. So during the night shift, it’s not available.’ –* participant I *‘It’s not a logical place for me [for the healthy vending machine]. Leaving the department is already a hassle. So the distance to the vending machine itself isn’t logical.’ –* participant J
		2. Facilities in or near the department	B and F	*‘We do have a microwave and such, so you can heat something up if you want to bring something. You have all the means to eat what you bring yourself. So I don’t think it [dietary habits] is negatively influenced in that sense.’ –* participant D
		3. Availability in the patient kitchen	B and F	*‘We have another refrigerator [in the patient kitchen] with milk, juice, yoghurt drink, chocolate milk, spreads, and also sweet toppings and bread, crackers, and crispbreads. Everything is there. In the freezer, there are pancakes and mini pancakes.’ –* participant T
		4. Treats from others	B	*‘And what is also difficult, is that patients often give us pastries and other sweets. They’re always stacked on the table.’ –* participant L
Policies	25	1. Policy and focus points of the organisation	B and F	*‘There isn’t really a policy, I think. […] They did once put out a bowl of nuts so everyone could have a handful during the night. That was about 2 or 3 years ago. But that was it, and we haven’t heard any feedback or anything since.’ –* participant R *‘I don’t think there is anything to be found about it [policy on diet at night], at least not that I know of. I haven’t actively searched for it, to be honest. But I don’t get the impression that they’re [the organisation] actively addressing it, you know.’ –* participant G
		2. Food supply by the organisation	B and F	*‘I know that the vending machine with healthy food is relatively new. So in that sense, I think there is at least an initiative to improve things. But when I look at our department and what’s available there, there are not many healthy options. So you’re really dependent on what you bring yourself. And if you forget [to bring food], then your choices are quite limited.’ –* participant F
		3. Provision of information by the organisation	B and F	*‘I think the organisation does try to spread knowledge about it [diet during night shifts], but they don’t provide sufficient motivation to employees to actually follow through.’ –* participant E *‘No, I have not received that information [about eating healthily during night shifts], no.’ –* participant F
Economic	24	1. Price plays no role	F	*‘We [at home] can afford healthy food, so we buy it. We prioritise it, but I think if you don’t have the money, it’s really difficult to make healthy choices.’ –* participant Q
		2. Looking for cheaper alternatives	F	*‘It might be a bit about the costs. Not that it would stop me from doing it [eating/buying healthy food], but it does make me adjust how I make my salad. For instance, if I think about using an avocado, which can be quite expensive, I might decide to skip it and go for cucumber or an egg instead, something simpler.’ –* participant U
		3. Price is a reason for other choices	B	*‘I buy more pre-packaged items for the night than during the day. That little salad. […] I find that quite expensive for the size you get. […] So I do think that if healthy food was cheaper, I’d be more inclined to buy it.’ –* participant N
Sociocultural	25	1. Influence of individual colleagues and their behaviour	B and F	*‘In the beginning, you tend to copy what colleagues do, but believe me, colleagues don’t always have the right lifestyle and examples to follow.’ –* participant A *‘In general I don’t think I have many colleagues who are big snackers, so that definitely helps. You know, seeing others eat can influence you, so that’s also a factor.’ –* participant V
		2. Eating together or alone	B and F	*‘I’m not sure why that is, but everyone eats at their own time, and that’s fine. It’s good too, because you really should maintain your own rhythm. So no, everyone does it [eating during the night shift] their own way.’ –* participant U *‘But I usually eat around 4–5 AM, we try to all eat together at that time.’ –* participant T
		3. General eating culture at night	B and F	*‘I have colleagues who arrive with three bags of chips in their bag and before you know it, the air fryer is out. There are also plenty of colleagues who eat healthily and have a similar eating pattern [as me].’ –* participant Q
		4. Discussing dietary intake	B and F	*‘We don’t really talk much about healthy eating at night. I’m not going to lecture someone about chips when I’m also eating them myself.’ –* participant S *‘Sometimes we ask each other how we manage it [diet at night], you know? Or if we’ve eaten that night and what we had. Just casual conversation. Then someone might say, “Well, I can never eat well during night shifts.” And another might eat either early or very late before they start working. Everyone has their own [pattern]’ –* participant V
		5. Colleagues bring food	B and F	*‘If people have something [snacks] with them, it’s easy to eat along while chatting.’ –* participant O *‘Then we agree: ‘You bring something one night, and I’ll bring something the next night.’ Sometimes it’s something unhealthy. We have a colleague who bakes fantastic cakes, so it’s great when someone brings cake. But others bring salads or make sandwiches.’ –* participant A
		6. Social influence at work	B	*‘There is a social aspect. I often don’t join in with everyone else when there’s a lot of tasty food around.’ –* participant Q
		7. Social influence at home	B and F	*‘My father also works night shifts; he also works at the hospital. He always takes bread and fruit with him, so I think I’ve picked up his approach, somewhat unconsciously. That’s how I started doing it too.’ –* participant M
Organisation of work	25	1. Work pressure and time	B and F	*‘Especially when it’s busy and you don’t have time to sit down quietly and eat your sandwich, then it’s easy to just grab and eat a biscuit, and then another biscuit.’ –* participant F *‘And when you’re doing nothing [at work], you get bored, and I think the easiest thing to do then during night shifts, is eat.’ –* participant C
		2. Tasks and responsibilities	B and F	*‘You can’t leave the department, you always have to stay behind the cameras. Sometimes a colleague comes to me, but it’s not on a regular basis. That makes it harder to really plan ahead and think consciously about what to bring [concerning food].’ –* participant O

#### Physical environment

Most participants mentioned the lack of available food in the organisation at night as a barrier to a healthy diet. This concerns both food provided for night workers in the department and options to purchase food themselves (e.g., at a supermarket or canteen). Even though they mentioned that a fruit basket was available in all departments, this basket was in most cases empty at the start of the night shifts. They also reported that a few healthy vending machines with vegan options (e.g., wraps or salads) were available in the hospital, but the participants mentioned the scarcity of these machines and their locations (e.g., too far from the worksite) as barriers. These machines were scarce and often located too far from the department. Moreover, vending machines offering unhealthy chocolate bars, crisps, and soft drinks were located in or near many departments. Other barriers in a few departments are the presence of a toaster, an air fryer, and an oven to prepare less healthy food options. The majority also mentioned treats (e.g., cakes, chocolate) that others bring in (e.g., patients, colleagues) as a barrier to healthy eating.

Some facilitators in the physical environment were mentioned. All departments had a fridge and microwave to store or heat up food from home, and coffee, tea, and water were available. Additionally, even though it was not officially allowed, the majority of the participants mentioned using the patient kitchen. This kitchen also provided healthy (e.g., fruit, bread, vegetables) as well as unhealthy (e.g., pancakes, grilled sandwiches) food.

#### Policies in the organisation

The majority of participants mentioned that there is no policy regarding nutrition during night shifts in the organisation. Some were aware of initiatives for night workers (e.g., providing nuts in the department), but these initiatives were organised locally or were temporary and not embedded. The limited supply of healthy food options by the organisation is another barrier mentioned by some participants. Moreover, a few night workers said that the provision of information by the organisation about a healthy diet during night shifts is limited or non-existent.

#### Economic environment

The majority of the participants indicated that the higher price of healthy food compared with unhealthy products did not affect their choices, while some others commented that healthy food options are expensive. Some of the latter group still made healthy choices, while seeking less expensive alternatives (e.g., frozen fruit instead of fresh fruit), and the others commented that they prefer to buy less expensive unhealthy snacks.

#### Sociocultural environment

Some participants mentioned that unhealthy dietary behaviours of their colleagues had a negative influence, as they imitated those behaviours or colleagues brought unhealthy snacks for sharing. Another barrier was a general unhealthy culture regarding diet during night shifts (e.g., having unhealthy snacks is seen as a sociable event) in some departments. Some participants reported that they never talked with colleagues about diet at night or they talked favourably about unhealthy behaviours (e.g., ‘snacking is a tradition’). Furthermore, a few mentioned that they experienced healthy eating as a disadvantage for social reasons, as they could not join colleagues who share their unhealthy food. Social influences at home were also a barrier, as living with a partner who supports unhealthy choices or living alone were mentioned by some participants.

The influence of dietary behaviours of colleagues could also be a facilitator, as some indicated that they copied the healthy dietary behaviours of colleagues, colleagues shared healthy food options, or they shared experiences with colleagues about specific healthy dietary choices. Moreover, a general culture regarding a healthy diet at night existed in some departments.

#### Organisation of work

Work pressure and specific tasks and responsibilities during night shifts had an impact on the dietary pattern of night workers. The majority reported having sufficient time for eating during night shifts, but some participants indicated that work pressure was sometimes too high to allow time for eating. Work pressure and specific tasks and responsibilities of the job also determined the timing of eating or whether it was possible to obtain or prepare food. In contrast, some participants mentioned that having a quiet shift increased boredom and therefore unhealthy food intake.

## Discussion

Night workers in the hospital are generally aware of the importance of healthy nutrition, but the current study showed that they make poorer dietary choices during night shifts than during other shifts. They lack knowledge on the timing and type of food consumption at night, and the attitude towards a healthy diet at night and the perception of self-efficacy in relation to performing healthy behaviours (i.e., motivation) are also important individual factors. Additionally, the physical and sociocultural work environment, the organisation of work and a lack of clear organisational policies play a critical role in dietary behaviours during night shifts.

Being awake and eating at night is desynchronised with the natural internal day-oriented circadian rhythms in our organs. This desynchronisation is a risk factor for sleep disorders^(19)^ and adverse metabolic consequences,^(5–8)^ which underlines the importance of creating optimal conditions for the body regarding timing and composition of food consumption among night workers. According to our model (Fig. 1), awareness is the first step at individual level towards healthy dietary behaviours. Awareness consists of three components, namely knowledge, cues to action, and risk perception, of which knowledge was found to be the most important factor for dietary behaviours. In line with a study among night workers in food services, healthcare, and industry in Ireland,^(15)^ our results showed that night workers generally have no or limited chrononutritional knowledge. The challenges to increasing knowledge are the lack of a clear and detailed consensus on the type and timing of food consumption at night and the insufficient distribution of these chrononutritional insights among night workers. Therefore, the scientific community is encouraged to reach a consensus on guidelines across countries and organisational settings. This could, for example, be achieved by conducting a Delphi study among chrononutrition experts, as was done previously for sleep hygiene guidelines.^(20)^ The second step is to translate these recommendations into practical guidelines targeting different occupational settings and distributed effectively among night workers and their employers.

As awareness is a pre-motivational stage in individual behaviour change models,^(17)^ being motivated to maintain a healthy diet might be difficult when knowledge on healthy dietary patterns is limited. This might explain our findings that various barriers in attitude and efficacy (i.e., motivation) influence dietary behaviours among night workers, such as the influence of diet at night on health and sleep, and the influence of needs and health status on diet at night. Motivation is an important factor to influence intention. This is shown by a meta-analysis that found associations between motivation factors and intention.^(21)^ Surprisingly, however, intention did not appear as either a facilitator or barrier in relation to healthy dietary behaviour at night in our study. This might be explained by the fact that intention is difficult to ask about explicitly during interviews and that our study focused on current dietary behaviour rather than the context in which behaviour change is promoted. Intention, in turn, is necessary for healthy dietary behaviours, according to our behaviour change model. However, this model assumes planned behaviour, and it is known that many individuals have intentions to perform healthy behaviours, but they fail to act on them for various reasons (i.e., the intention-behaviour gap).^(22)^ For example, impulsive or spontaneous behaviour that is the result of automatic processes may counteract the intention to perform healthy behaviours.^(23)^ This suggests that planned behaviour is not sufficient for behaviour change and that environmental factors are important for healthy dietary behaviours to limit the influence of, for example, impulses.

Indeed, our study showed that environmental factors also play a crucial role in healthy behaviours. In line with previous research in Ireland on barriers and facilitators to healthier lifestyle,^(15)^ the physical and sociocultural environmental factors and the factors within the organisation of work were found to be important at the environmental level. A possible explanation for the significance of the physical and sociocultural environment and the organisation of work is that individual healthy dietary behaviours are easier when the conditions at the environmental level are supportive (e.g., availability of healthy food, a healthy sociocultural environment, balanced work pressure). Concerning the physical environment, previous studies from United States of America and South-Africa showed that the availability of healthy food at the workplace is an important facilitator for healthy dietary behaviours of healthcare workers, irrespective to their work schedule,^(24,25)^. These findings are in line with our study that focused on night shift only. Regarding the sociocultural environment, a qualitative study among night workers with diabetes in the United Kingdom found results similar to our study.^(26)^ Examples from that study include workers being unable to resist sweets brought in by patients and visitors, and colleagues influencing food choices,^(26)^ which were also found in the current study as environmental factors that stimulate impulsive behaviours. Concerning the organisation of work, limited evidence of the effects of working conditions on diet quality exists,^(27)^ and future studies should provide insights into how working conditions (e.g., working hours, physical conditions, psychosocial factors) influence diet at night. The lack of clear organisational policies and guidelines regarding nutrition among night workers might be the underlying cause of conditions in the work environment that fail to support the study population, such as the unavailability of healthy food options during the night. To date, most intervention studies among night workers aiming at dietary changes have focused on the individual level (e.g.,^(28)^) or on providing healthy food options at the workplace (e.g.,^(29,30)^), whereas it would be recommended to incorporate the environmental context to a larger extent in interventions.^(31)^ We recommend future research to gain insight into how the influential individual and environmental factors could be translated into effective interventions and implementation practices.

### Strengths and limitations

To our knowledge, this study is the first of its kind that has qualitatively investigated the factors underlying healthy and unhealthy dietary behaviours of night workers in the healthcare within the Netherlands. The theory-based and qualitative approach in which an extensive integrated behaviour change model was used to gain a comprehensive overview of individual and environmental factors is an important strength. A second strength is that two researchers conducted the coding and they consulted with a third researcher, if necessary, which improved coding quality.

Several limitations should also be considered. First, questioning the dietary behaviours of night workers retrospectively was challenging, as the information provided was more general, concerning an average night shift rather than specific shifts. Gathering nuanced information was therefore challenging, as participants found it difficult to recall details about specific night shifts. To gain more specific insight into the dietary habits of night workers, daily diaries for a sequence of night shifts are recommended in future research. Second, no information on socioeconomic position, ethnicity or health status of participants was gathered, which could have caused a ‘selection bias’. However, our sampling approach was inclusive on ‘hospital’ level promoting inclusivity. Third, it was difficult to identify some factors of the integrated behaviour change model, although these could play an important role in dietary behaviours. For example, it might be difficult for participants to comment on their risk perception of unhealthy dietary behaviours if not asked explicitly. Another example is intention, since addressing the different phases of intention explicitly is difficult to incorporate into questions in interviews, as the intention phase can be detected based on different statements and views. General observations from the interviews indicate that only a few participants had no or a low intention to change their dietary behaviour, while most showed the intention to create or maintain a healthy dietary pattern at night.

## Conclusion

The current study findings reveal that individual awareness about healthy dietary patterns during night shifts, motivation to perform healthy behaviours, physical and sociocultural environment, organisation of work, and organisational policies are important factors to address for future implementation of dietary interventions among night workers. Concerning the individual factors, reaching a consensus on the timing and composition of food intake during night shifts and providing workers and employers with this information are important to improve their awareness. Regarding environmental factors, organisational policies that focus on the social culture, working conditions, and availability of affordable or free healthy food options at night are crucial for stimulating favourable individual factors, to ensure a change in individual behaviour towards healthy behaviours.
